# The complete mitochondrial genome of *Belzebub intermedius* (Decapoda, Dendrobranchiata, Luciferidae)

**DOI:** 10.1080/23802359.2018.1473718

**Published:** 2018-05-17

**Authors:** Seong-Yun Ju, Ji-Hun Song, Seung-Min Lee, Gi-Sik Min

**Affiliations:** a Department of Biological Sciences, Inha University, Incheon, South Korea;; b West Sea Fisheries Research Institute, National Institute of Fisheries Science, Incheon, South Korea

**Keywords:** *Belzebub*, complete mitogenome, Decapoda, Luciferidae, Sergestoidea

## Abstract

We determined the mitochondrial genome (mitogenome) sequence of *Belzebub intermedius* (Hansen, 1919). To the best of our knowledge, this is the first complete mitogenome in the family Luciferidae. The complete mitogenome of *B. intermedius* is 16,001 bp in length, with 13 protein-coding genes (PCGs), 22 transfer RNAs (tRNAs), two ribosomal RNAs (rRNAs), and a control region (CR). The gene arrangement of *B. intermedius* is almost identical to that of the same sergestoid species, *Acetes chinensis* Hansen, 1919, except that there is no additional *trnS1*. A maximum-likelihood tree, constructed using 18 decapod mitogenomes, confirmed that *B. intermedius* occupied the most basal position within the suborder Dendrobranchiata.

Dendrobranchiata, a suborder of the decapod shrimps (commonly known as prawns), comprises two superfamilies, Sergestoidea and Penaeoidea. The Sergestoidea is divided into two families, Luciferidae and Sergestidae. The Luciferidae was considered monotypic with only the genus *Lucifer*. However, *Belzebub* Vereshchaka, Olesen & Lunina, 2016 has been established as a new genus through morphological cladistic analysis. Therefore, Luciferidae is currently divided into two genera (Vereshchaka et al. 2016). The *Belzebub* consists of five species, of which *B. intermedius* is considered a euryhaline and a subtropical pelagic species with adaption to high temperatures (Xu 2010; WoRMS 2018). Furthermore, the distribution and abundance of *B. intermedius* are known to affect the weather, global warming, and the Kuroshio currents in both of the East China Sea and the Yellow Sea (Ma et al. 2009). In the present study, we determined the complete mitogenome of *B. intermedius*. To the best of our knowledge, this is the first complete mitogenome in Luciferidae. Therefore, it would be useful and essential for further studies on phylogenetic relationships and the evolution of Dendrobranchiata, including Sergestoidea.

A single specimen of *B. intermedius* was collected using a Norpac net from the Eulwang-ri beach (37°26′N, 126°22′E) in the Yellow Sea, South Korea. Specimen was deposited in the National Institute of Biological Resources, Incheon, South Korea (specimen deposit no. NIBRIV0000816436). Mitochondrial DNA extraction, sequencing and gene annotation were performed according to the methods described by Song et al. (2016). Commercial software Geneious version 6.1.3 (Biomatters Ltd, Auckland, New Zealand) was used for *de novo* assembly of raw reads and producing a circular form of the complete mitogenome with average coverage. The gene annotation was carried out using the MITOS (Bernt et al. 2013) and ARWEN (Laslett and Canbäck 2008). A phylogenetic tree was constructed using the software MEGA7.0 by the maximum-likelihood method (Kumar et al. 2016).

The complete mitogenome of *B. intermedius* (GenBank accession no. MG719343) is a circular molecule of 16,001 bp in length, with 13 protein-coding genes (PCGs), 22 transfer RNAs (tRNAs), two ribosomal RNAs (rRNAs), and a control region (CR). The gene content and arrangement of *B. intermedius* are almost identical to those of the same sergestoid species *A. chinensis*, except that there is no additional *trnS1* (see Kim et al. 2012 for information of additional *trnS1*).

To infer the phylogenetic relationships, we performed a maximum-likelihood analysis using the concatenated sequences of 13 PCGs from 18 complete mitogenomes of decapods, including two sergestoid and 13 penaeoid species. Furthermore, three species of the suborder Pleocyemata were used as outgroups. As a result, *B. intermedius* occupied the most basal position within Dendrobranchiata (Figure 1).

**Figure 1.**
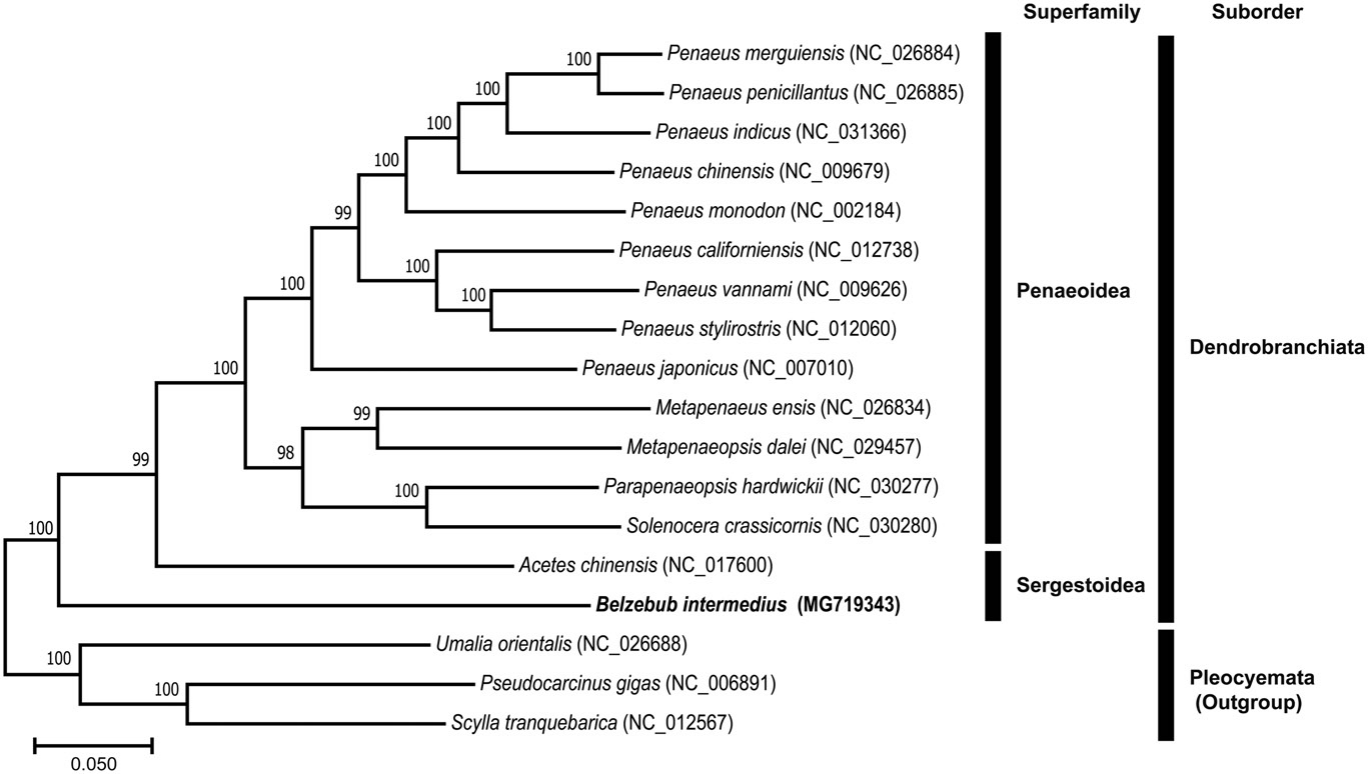
Maximum-likelihood (ML) tree based on the mitogenome of *B. intermedius* with 17 other decapod species was constructed by using MEGA7 with 1000 bootstrap replicates. GenBank accession numbers are provided following species names. The bootstrap supports are shown in each node.
